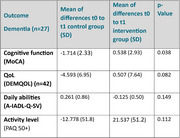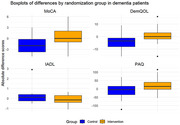# Preliminary Findings from a 12‐Week multidomain app‐based Intervention to enhance cognitive Function in Dementia Patients: Results from the MEMODIO_APP@CARE Randomized Control Trial

**DOI:** 10.1002/alz70860_103568

**Published:** 2025-12-23

**Authors:** Gereon Nelles, Felix Bicu, Valentina Weil, Tiemo Steinmann, Doron Benjamin Stein, Alicia Bicu, Arnim Quante, Maria Cristina Polidori

**Affiliations:** ^1^ Neuromed Campus, Cologne, Nordrhein‐Westphalen, Germany; ^2^ memodio GmbH, Potsdam, Brandenburg, Germany; ^3^ University of Heidelberg, Medical Faculty Mannheim, Mannheim, Baden‐Württemberg, Germany; ^4^ Friedrich von Bodelschwingh Klinik, Berlin, Berlin, Germany; ^5^ University Hospital of Cologne, Köln, Cologne, Germany

## Abstract

**Background:**

The growing prevalence of dementia in aging populations underscores the urgent need for effective prevention and intervention strategies. The Lancet Commission on Dementia highlights the role of targeted evidence‐based lifestyle modifications in reducing the risk or delaying the progression of cognitive impairment. Given the limitations of pharmacological treatments, there is an increasing need for non‐pharmacological, accessible interventions. To meet this need, the MEMODIO app offers a multidomain therapeutic approach tailored to individuals with mild cognitive impairment (MCI) or mild dementia.

**Objective:**

To evaluate the potential of an app‐based therapy in improving functional outcomes for individuals with MCI and mild dementia. This interim analysis focuses on preliminary findings from the dementia study arm, comprising 27 participants, with data collected up to November 2024.

**Method:**

To evaluate the potential of an app‐based therapy in improving functional outcomes for individuals with MCI and mild dementia. This interim analysis focuses on preliminary findings from the dementia study arm, comprising 27 participants, with data collected up to November 2024.

**Result:**

In the Dementia group (*n* = 27), the average age was 81 years, with 13 female participants out of 27. The IG showed a significant improvement in cognitive performance, as reflected in the MoCA score (‐1.71±2.3 in SoC vs. 0.54±2.9 in IG, *p* = 0.038). Although quality of life, physical activity, and activities of daily living did not show statistically significant changes at this interim stage, all three outcomes exhibited a positive trend in the IG relative to baseline.

**Conclusion:**

This interim analysis demonstrated that the use of the MEMODIO app significantly improved cognitive function in dementia patients compared to standard care alone. Ongoing analyses aim to provide further insights into additional outcomes and the long‐term impact of the intervention.